# rTMS over bilateral superior temporal gyri combined with auditory-speech training for post-stroke pure word deafness: a case report

**DOI:** 10.3389/fpsyg.2026.1911332

**Published:** 2026-08-10

**Authors:** Xinxin Chen, Yichen Chang, Yan Leng, Xi Chen, Yongxue Li

**Affiliations:** Department of Rehabilitation Medicine, The First Affiliated Hospital, Sun Yat-sen University, Guangzhou, China

**Keywords:** auditory-speech training, case report, pure word deafness, repetitive transcranial magnetic stimulation, superior temporal gyrus

## Abstract

**Objective:**

To describe the application of high-frequency rTMS over the bilateral superior temporal gyri (STG) combined with auditory-speech training and assess its effects on auditory comprehension.

**Methods:**

A 54-year-old woman with PWD secondary to bilateral temporal ischemic strokes underwent a 10-day combined intervention. She received 10 Hz rTMS over the left STG, followed by auditory-speech training, and then received rTMS over the right STG with identical parameters. Treatment outcomes were assessed using the Western Aphasia Battery (WAB), the Psycholinguistic Assessment in Chinese Aphasia (PACA), the Boston Naming Test (BNT), and the abbreviated Token Test.

**Results:**

Audiological evaluation confirmed the patient’s normal peripheral hearing. At baseline, the patient had preserved cognitive function but severe auditory comprehension deficits. On the WAB, the Aphasia Quotient was 40.8 and the Cortical Quotient was 56.47. The abbreviated Token Test score was 3.5/36, and the BNT correct rate was 83.3%. Environmental sound recognition, reading, naming, oral reading, picture-based writing, and direct copying were at or near ceiling levels. Baseline performance on PACA was severely impaired. After the 10-day intervention, the patient’s WAB Aphasia Quotient improved to 65.9, Token Test scored to 16/36, and BNT correct rate remained 83.3%. On PACA, notable improvements were observed in phoneme discrimination (4/30 to 19/30), minimal difference word judgment (5/40 to 32/40), speech input buffer (1/24 to 6/24), speech input lexicon (3/24 to 12/24), auditory word-picture matching (13/40 to 32/40), auditory semantic knowledge (0/36 to 20/36), word repetition (0/30 to 10/30), word repetition with oral-cue prompting (6/30 to 15/30), and dictation (0/40 to 17/40). No adverse events occurred.

**Conclusion:**

This case provides preliminary evidence that combined bilateral STG rTMS and auditory-speech training may be feasible and may support auditory-verbal rehabilitation in PWD. Controlled studies with longer-term follow-up are needed.

## Introduction

Pure word deafness (PWD), also termed auditory verbal agnosia, is a rare cortical auditory disorder after ischemic strokes characterised by a selective impairment in the comprehension and repetition of spoken language, while spontaneous speech, reading, writing, and the perception of environmental sounds remain relatively preserved (Buchman et al., 1986; Slevc and Shell, 2015). Standard pure-tone audiometry of PWD is typically normal, thereby ruling out peripheral hearing loss. PWD is characterised by preservation of spontaneous speech and environmental sound perception, which distinguishes it from cortical deafness (complete loss of all sound perception), generalised auditory agnosia (impaired recognition of both speech and non-speech sounds), and Wernicke’s aphasia (fluent paraphasic jargon, poor auditory comprehension, and impaired repetition, reading, and writing) (Miceli and Caccia, 2022; Miceli and Caccia, 2023). According to the dual-stream model, the dorsal superior temporal gyrus (dSTG) bilaterally supports early spectrotemporal analysis, from which information diverges into two pathways, including a ventral stream mapping sound to meaning via the STS and MTG, and a dorsal stream supporting auditory-motor integration (Hickok and Poeppel, 2007). Wernicke’s area, located in the left posterior STG, maps phonological information onto lexical representations. Damage to Wernicke’s area produces the classical features of Wernicke’s aphasia, whereas PWD arises from a deficit upstream of this region.

PWD arises from a deficit upstream of Wernicke’s area, specifically at the level of phonetic analysis within the bilateral STG. The neuroanatomical hallmark of PWD is bilateral damage to the dSTG or its underlying white matter connections, which severs the transmission of auditory–phonetic information to Wernicke’s area while leaving its internal linguistic operations intact (Geschwind, 1965; Poeppel, 2001). The preservation of environmental sound perception in PWD indicates that the primary auditory cortex (Heschl’s gyrus, Brodmann area 41) is typically spared. The core deficit lies at the level of phonetic decoding in the bilateral dSTG rather than in early acoustic registration (Miceli and Caccia, 2023). Sequential bilateral temporal infarctions often lead to persistent PWD, as the unaffected hemisphere loses its compensatory function after the second stroke (Virvidaki et al., 2021; Casilio et al., 2024). Despite well-documented clinical descriptions of PWD (Gutschalk et al., 2015; Maffei et al., 2017), treatment options remain extremely limited. Conventional speech-language therapy alone yields little benefit, because auditory perception remains severely impaired even after prolonged training (Silva et al., 2020; Slotwinski et al., 2020). One study reported some improvement with specialised phoneme-processing training (Tessier et al., 2007), but this finding has not been replicated. Although repetitive transcranial magnetic stimulation (rTMS) has shown promise in improving auditory comprehension in post-stroke aphasia (Lefaucheur et al., 2014; Arheix-Parras et al., 2021), evidence for rTMS specifically in PWD is exceedingly scarce.

Here we report a 54-year-old woman with PWD after two sequential bilateral temporal strokes. She had fluent spontaneous speech, normal environmental sound recognition and pure-tone audiometry, but severe impairment in spoken language comprehension, repetition and dictation. Naming, reading and writing were relatively preserved, and bilateral temporal MRI lesions confirmed the PWD diagnosis. The patient had received no speech-language intervention for over 3 years. Using the Psycholinguistic Assessment in Chinese Aphasia (PACA) (Wang et al., 2013), she showed severe impairment beginning at the auditory analysis module (except environmental sounds), indicating a failure at the phonetic-analysis level consistent with bilateral dorsal superior temporal gyrus (dSTG) dysfunction. Because bilateral dSTG damage is the core pathology of PWD and the dSTG serves as the essential auditory-phonological gateway, we targeted the bilateral dSTG with 10 Hz rTMS combined with auditory-speech training. At presentation, she underwent a 10-day combined treatment. Global cognitive function was assessed using the Mini-Mental State Examination (MMSE) (Truong et al., 2024) and the Montreal Cognitive Assessment (MoCA) (Islam et al., 2023). Language function was evaluated with the Western Aphasia Battery (WAB) (Turkstra, 2011; Li et al., 2022), and the level of auditory processing impairment was analysed using the PACA (Wang et al., 2010; Li et al., 2015). Naming ability was assessed with the Boston Naming Test (BNT) (Olabarrieta-Landa et al., 2024; Li et al., 2025), and auditory comprehension with the shortened Token Test (Julio-Ramos et al., 2024; Lin et al., 2014). PWD is a highly disabling condition that severely impairs daily communication and quality of life, yet its rarity and poor response to conventional therapy pose major clinical challenges. To our knowledge, this is the first systematic description of a combined bilateral rTMS and auditory-speech training treatment for PWD.

## Case description

A 54-year-old, right-handed woman with 9 years of education presented to our Department of Rehabilitation Medicine on March 27, 2026. The patient had previously worked in a garment factory. She reported fluent spontaneous speech but a complete inability to understand spoken language. This deficit had persisted for over 3 years. Due to her comprehension deficit, she was unable to understand conversations with family members, could not follow verbal instructions in daily activities, and was unable to use the telephone or watch television. These difficulties severely limited her social participation and required her family to communicate with her primarily through writing or gestures. Peripheral hearing was normal. In June 2022, she suffered an acute right temporal lobe ischemic stroke due to cerebral embolism. She underwent arterial thrombolysis and mechanical thrombectomy at an outside hospital, with good initial recovery. In September 2022, she experienced a second ischemic stroke involving the left temporal lobe. The presenting symptoms were sudden altered consciousness and disorganized speech. After this second stroke, the patient developed a persistent and profound deficit in spoken language comprehension. The patient’s spontaneous speech remained fluent and well-articulated. She had no history of hypertension, hyperlipidemia, or drug allergies.

**Figure 1:**
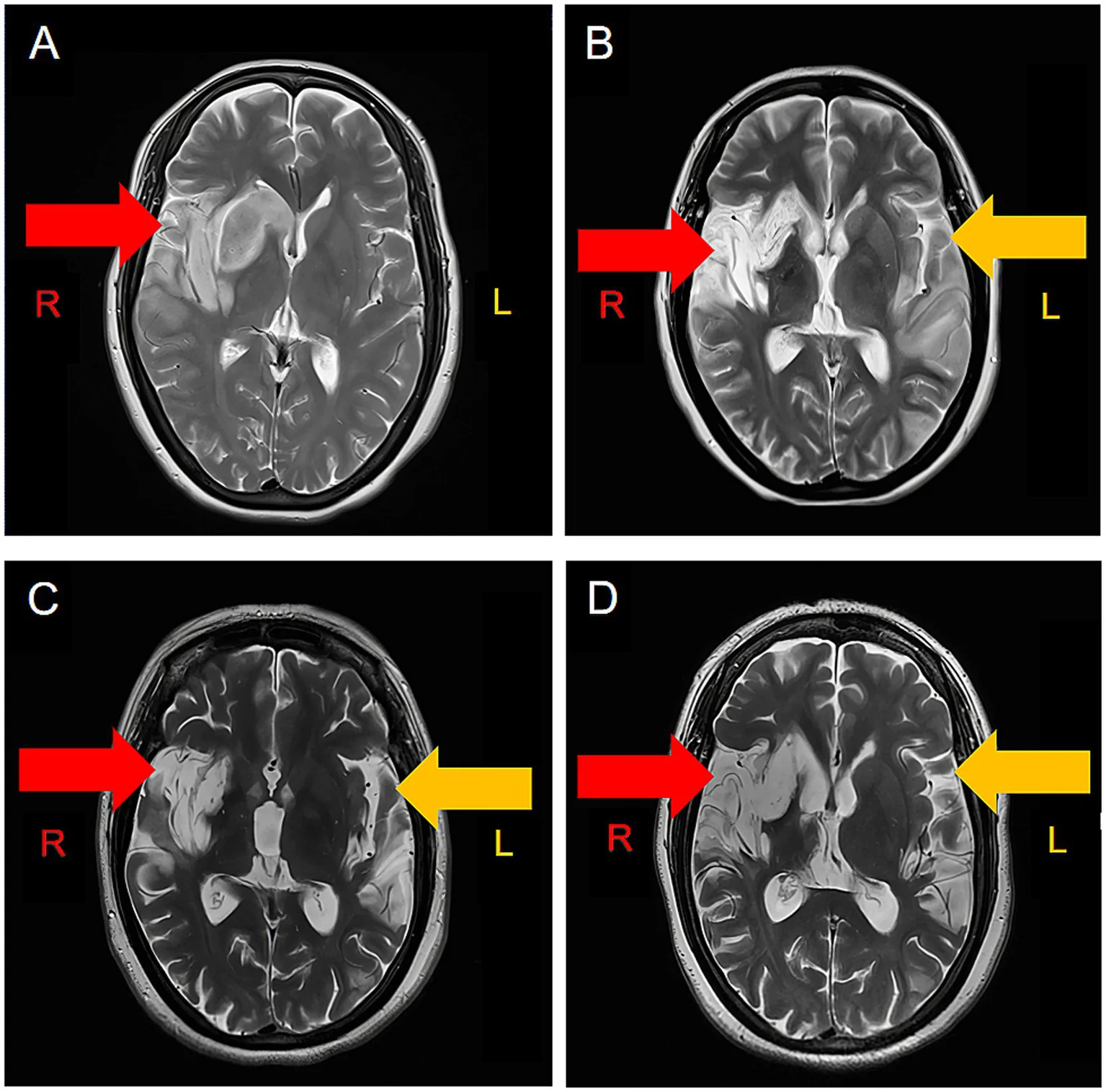
Serial brain MRI scans of the patient. Red “R” on the left and yellow “L” on the right mark hemispheric sides. Red arrows point to right temporal lesions; yellow arrows to left temporal lesions. **(A)** June 7, 2022: Post-acute changes of right-hemispheric infarction involving the frontal, temporal, and insular lobes, the basal ganglia, and the parietal lobe. There is partial resolution of the previously noted mildly hyperdense lesion in the right basal ganglia. No restricted diffusion is seen. Diagnosis: Post-acute ischemic changes in the right hemisphere. **(B)** September 2, 2022: The left temporal-insular-occipital region shows confluent areas of T1 hypointensity and T2 hyperintensity, with corresponding hyperintensity on T2-FLAIR and DWI, hypointensity on ADC, and effacement of the adjacent sulci-consistent with acute infarction. In addition, bilateral frontal-temporal-insular lobes and the right corona radiata-basal ganglia region demonstrate well-demarcated, patchy abnormal signals, representing remote infarcts with encephalomalacia. Diagnosis: Acute left temporal-insular-occipital infarction; remote infarcts with encephalomalacia in bilateral frontal-temporal-insular lobes and the right corona radiata-basal ganglia region. **(C)** May 23, 2024: Multiple well-circumscribed patchy signal abnormalities are present in bilateral frontal-temporal-insular lobes, the right corona radiata-basal ganglia, the right parietal lobe, and the left occipital lobe. No restricted diffusion or mass effect is noted. Mild ventriculomegaly and slight widening of the cortical sulci and Sylvian fissures are observed. Diagnosis: Multiple remote infarcts with partial encephalomalacia; leukoaraiosis; mild brain atrophy. **(D)** April 2, 2026: The right basal ganglia, insula, and bilateral frontal-temporal lobes show irregular, patchy T1 hypointensity and T2 hyperintensity with a characteristic FLAIR appearance -central low signal rimmed by peripheral high signal, indicative of encephalomalacia with surrounding gliosis. Scattered punctate foci of T2/FLAIR hyperintensity (ischemic foci) are noted in bilateral frontal–parietal lobes, periventricular white matter, and both cerebellar hemispheres. There is symmetrical enlargement of the lateral ventricles and basal cisterns, with deepened and widened cortical sulci and fissures. Diagnosis: Encephalomalacia with reactive gliosis in bilateral frontal-temporal lobes, basal ganglia, and insula; scattered ischemic infarcts (some with small-foci encephalomalacia); moderate brain atrophy.

The patient underwent serial brain MRI over a follow-up period of 3 years and 7 months, which showed progressive evolution of bilateral temporal lobe lesions (Figure 1). The patient’s most recent scan, performed in April 2026, demonstrated encephalomalacia with surrounding gliosis in the bilateral frontal-temporal lobes, basal ganglia, and insula, together with brain atrophy (Figure 1D). The patient also underwent audiological evaluation in January 2026, including otolaryngological examination and pure-tone audiometry, both of which yielded entirely normal findings, confirming intact peripheral hearing.

On cognitive screening, the patient scored 28/30 on the MMSE and 25/30 on the MoCA, indicating that the patient’s global cognitive function remained largely intact apart from language-related domains. The patient’s activities of daily living were fully independent (modified Barthel Index: 100/100). On the WAB, she obtained an Aphasia Quotient of 40.8 and a Cortical Quotient of 56.47, with relatively preserved spontaneous speech fluency, naming, reading, writing, construction, visuospatial function, and calculation, but severely impaired auditory comprehension and repetition. The patient’s correct response rate on the BNT was 83.3%. The PACA further confirmed the patient’s profound deficits in auditory comprehension, repetition, and dictation, while reading, naming, oral reading, picture-based writing, and direct copying were largely spared. The patient scored 3.5/36 on the abbreviated Token Test, consistent with an extremely severe auditory comprehension deficit. All evaluation results at baseline are summarized in Table 1.

**Table 1:** Cognitive and language assessments before and after treatment.

Assessment	Domain	Pre-treatment	Post-treatment
MMSE	Total (Score/30)	28	29
	Orientation (Score/10)	9	10
	Registration (Score/3)	3	3
	Attention and Calculation (Score/5)	5	5
	Recall (Score/3)	3	3
	Language (Score/9)	8	8
MoCA	Total (Score/30)	25	26
	Visuospatial/Executive (Score/5)	4	4
	Naming (Score/3)	3	3
	Attention (Score/6)	5	5
	Language (Score/3)	0	1
	Abstraction (Score/2)	2	2
	Delayed Recall (Score/5)	5	5
	Orientation (Score/6)	6	6
WAB	Aphasia Quotient (/100)	40.8	65.9
	Cortical Quotient (/100)	56.47	75.73
	Information Content (/10)	1	5
	Fluency (/10)	9	10
	Auditory Comprehension (/10)	1.4	4.95
	Repetition (/10)	0	3.6
	Naming (/10)	9	9.4
	Reading (/10)	10	10
	Writing (/10)	10	10
	Praxis (/10)	6.67	8.83
	Construction, Visuospatial, Calculation (/10)	10	10
BNT	Correct (/30)	25	25
	Correct Rate (%)	83.3	83.3
PACA			
Auditory comprehension	Phoneme Discrimination (/30)	4	19
	Minimal Difference Word Judgment (/40)	5	32
	Speech Input Buffer (/24)	1	6
	Speech Input Lexicon (/24)	3	12
	Environmental Sound Recognition (/24)	23	24
	Auditory Word–Picture Matching (/40)	13	32
	Auditory Semantic Knowledge (/36)	0	20
Reading	Visual Word–Picture Matching (/40)	40	40
	Visual Semantic Knowledge (/36)	36	36
	Word Categorization (/36)	36	36
Naming	Picture Categorization (/36)	36	36
	Oral Picture Naming (/40)	40	40
Repetition	Word Repetition (/30)	0	10
	Word Repetition with Oral-Cue Prompting (/30)	6	15
Oral reading	Word Reading Aloud (/40)	40	40
Writing	Dictation (/40)	0	17
	Picture-Based Writing (/40)	40	40
	Direct Copying (/30)	30	30
Abbreviated token test	Score (/36)	3.5	16

MMSE, Mini-Mental State Examination; MoCA, Montreal Cognitive Assessment; WAB, Western Aphasia Battery; BNT, Boston Naming Test; PACA, Psycholinguistic Assessment in Chinese Aphasia.

These clinical and neuroimaging findings were consistent with a diagnosis of pure word deafness. Her selective impairment of speech comprehension, repetition, and dictation occurred in the setting of normal peripheral hearing, preserved environmental sound recognition, and relatively intact spontaneous speech, reading, and writing, while serial brain MRI confirmed progressive bilateral temporal lobe lesions as the underlying pathology. She had received no speech-language therapy at any time during the more than 3 years since symptom onset. She was therefore enrolled in a 10-day combined protocol of bilateral rTMS and auditory-speech training, as detailed below.

## Methods

### rTMS protocol

rTMS was delivered using a CCY-II magnetic stimulator (Yiruide Corporation, Wuhan, China) equipped with a figure-of-eight coil. Bilateral stimulation was applied over the left and right superior temporal gyrus (STG). Coil positioning was guided by the international 10–20 EEG system: the left STG was targeted at the midpoint between T3 and C3 (approximately corresponding to Brodmann area 22), and the right STG at the midpoint between T4 and C4. The patient’s resting motor threshold (RMT) was defined as the minimum stimulator output intensity required to elicit a visible thumb twitch in the contralateral hand in at least 5 of 10 consecutive trials. For each hemisphere, stimulation was delivered at 10 Hz with an intensity of 80% of the patient’s RMT. Each session delivered 800 pulses per hemisphere, consisting of 20 trains of 40 pulses each, with a 26-s inter-train interval. At 10 Hz, each 40-pulse train lasted 4 s. Including the inter-train intervals and brief rest periods, the total session time per hemisphere was 10 min. The treatment sequence was as follows: left STG rTMS first, immediately followed by a 60-min inter-hemispheric interval, which included 30 min of auditory-speech training and 30 min of rest. Then right STG rTMS was administered using identical parameters. The protocol was applied for 10 consecutive days. The stimulation parameters were selected based on established safety guidelines and previous rTMS studies in post-stroke aphasia (Tan et al., 2024; Rossi et al., 2021). Ten Hertz and 800 pulses per hemisphere were chosen based on standard excitatory protocols used in post-stroke language rehabilitation (Tan et al., 2024), and 80% of resting motor threshold was selected to remain well within safety limits (Rossi et al., 2021). Bilateral stimulation was chosen because the patient had sequential bilateral temporal lesions, and PWD is characterized by bilateral STG dysfunction that isolates Wernicke’s area from phonetic input. As supported by previous dual-hemisphere rTMS studies in aphasia, bilateral protocols may yield superior language outcomes when the left hemisphere is severely compromised (Li et al., 2025). The left hemisphere was stimulated first due to its predominant role in phonetic and phonological encoding, and the 60-min interval between hemispheres allowed auditory-speech training to be delivered during this period.

After each session, the patient was systematically asked about potential adverse effects, including headache, scalp discomfort, dizziness, fatigue, mood changes, tinnitus, and any auditory symptoms. General observation for seizure-like symptoms was also performed. No adverse events were reported during or after any session.

### Auditory-speech training

Auditory-speech training was conducted in a one-on-one setting using auditory stimuli as the sole input. The training encompassed four hierarchical levels, including detection, discrimination, identification, and comprehension, each designed with a specific rehabilitative rationale. Auditory detection targeted basic sound awareness and orienting to auditory stimuli. Auditory discrimination, including phoneme discrimination and minimal word pair judgment, targeted phonetic contrast processing at the prelexical level, specifically the ability to distinguish between similar speech sounds. Auditory identification, including auditory word-picture matching, aimed to establish sound-to-meaning mapping by supporting the association of acoustic-phonetic information with phonological and lexical representations. Auditory comprehension, including auditory semantic knowledge tasks and yes/no questions, promoted higher-level auditory-verbal integration and semantic access. The training primarily aimed to restore impaired auditory-phonetic processing through bottom-up auditory input. Written words and pictures were used only as response options to indicate the patient’s choice, not as visual cues to bypass auditory processing. Immediate feedback was provided after each response. Task difficulty was adaptively adjusted using a two-up/two-down rule, meaning two consecutive correct responses increased difficulty whereas two consecutive errors decreased it. Progression to the next level required ≥80% accuracy across sessions. All training stimuli were drawn from separate lists distinct from those used in the PACA and other assessments. Each session consisted of approximately 40–60 trials per hierarchical level. Stimuli included syllables (for detection and discrimination tasks), single-character words (for identification), and phrases or simple sentences (for comprehension). Lexical tones were specifically trained within the phoneme discrimination and minimal word pair judgment tasks. Task difficulty was adjusted along multiple dimensions, including stimulus intensity, inter-stimulus interval, array size, and linguistic complexity, using a two-up/two-down rule. Representative examples of the tasks used at each level are provided in Supplementary Table S2.

### Outcome measures

**Figure 2:**
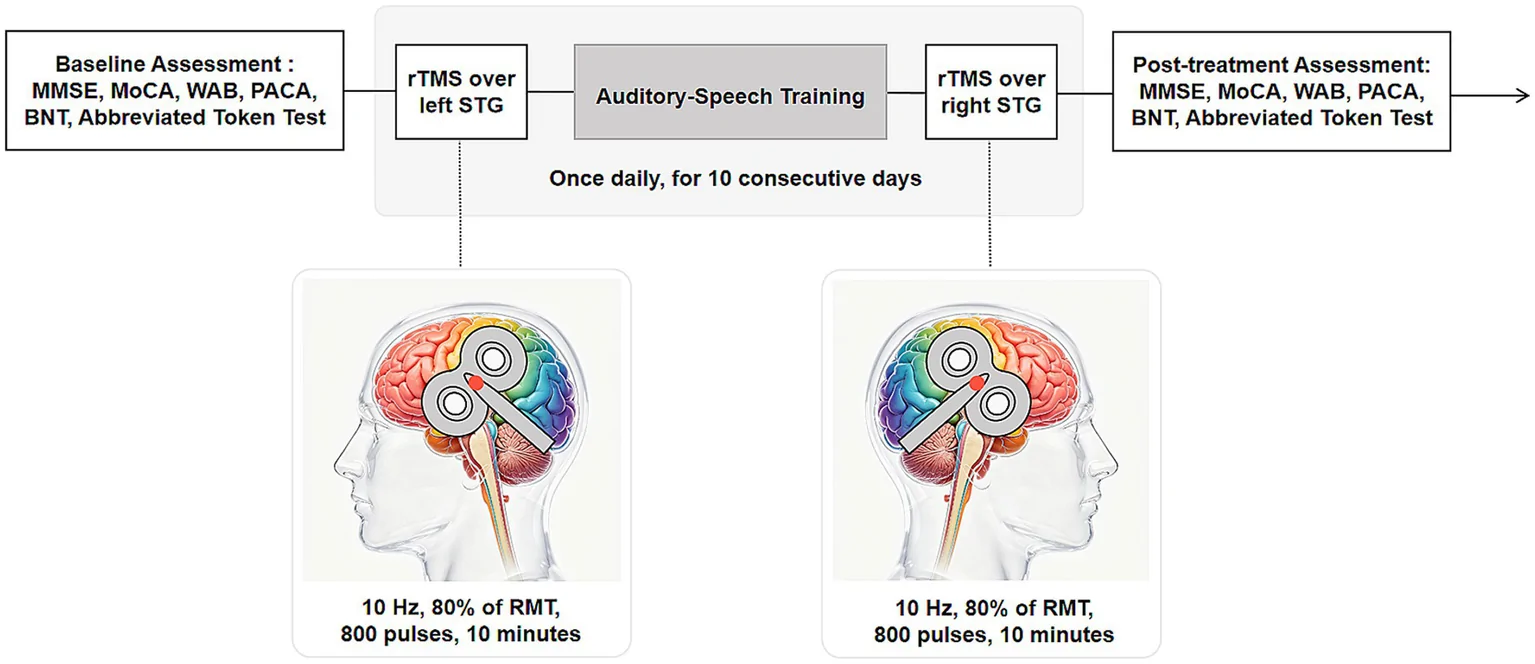
Study design and treatment protocol. MMSE, Mini-Mental State Examination; MoCA, Montreal Cognitive Assessment; WAB, Western Aphasia Battery; PACA, Psycholinguistic Assessment in Chinese Aphasia; BNT, Boston Naming Test; repetitive transcranial magnetic stimulation, rTMS; STG, superior temporal gyrus; RMT, resting motor threshold.

Outcomes were assessed at baseline and 2 days after completion of the 10-day intervention. Cognitive function and activities of daily living were measured with the MMSE, MoCA, and modified Barthel Index (MBI). Language function was evaluated using the WAB to obtain an overall language profile. To provide a modular, pathway-based evaluation of auditory comprehension, the PACA was employed. The PACA assesses multiple modules within the auditory input processing pathway, including sound perception, auditory analysis, speech input buffer, speech input lexicon, and lexical-semantic routes. Specifically, the auditory comprehension domain comprises phoneme discrimination, spoken word matching with minimal differences, environmental sound recognition, auditory word-picture matching, and auditory semantic knowledge. The reading domain includes visual word-picture matching, visual semantic knowledge, and word categorization. The naming domain consists of picture categorization and oral picture naming. The repetition domain covers word repetition and word repetition with oral-cue prompting. The oral reading domain involves word reading aloud. Finally, the writing domain includes dictation, picture-based writing, and direct copying. Representative examples of each PACA domain are provided in Supplementary Table S1. Naming was further assessed with the BNT, and auditory comprehension severity was measured with the abbreviated Token Test. The study design and treatment protocol are shown in Figure 2.

### Statistics

This is a single-case observational study and no inferential statistical analysis was performed. Changes in scale scores were described qualitatively and quantitatively.

## Results

The patient completed all 10 sessions of the combined bilateral rTMS and auditory-speech training protocol without any adverse events. Treatment outcomes assessed at baseline and 2 days after completion of the 10-day intervention are summarized in Table 1.

Global cognitive function remained largely stable, with her MMSE score increasing from 28 to 29 and her MoCA score from 25 to 26.

Overall language function, as measured by the WAB, showed a marked improvement. Her Aphasia Quotient increased from 40.8 to 65.9, and her Cortical Quotient rose from 56.47 to 75.73. Among the WAB subtests, the most notable gains were observed in auditory comprehension (from 1.4/10 to 4.95/10), repetition (from 0/10 to 3.6/10), and information content (from 1/10 to 5/10). Praxis also improved, increasing from 6.67/10 to 8.83/10. The remaining subtests were already near ceiling at baseline and remained essentially unchanged after treatment, including fluency, naming, reading, writing, and the combined construction, visuospatial, and calculation subtest.

The patient’s BNT correct rate remained 83.3% (25/30). The abbreviated Token Test score increased from 3.5/36 to 16/36, reflecting a shift from a profound to a severe auditory comprehension deficit.

Module-by-module analysis of the auditory input pathway using PACA revealed improvements across multiple domains. Within the auditory comprehension domain, phoneme discrimination increased from 4/30 to 19/30, minimal difference word judgment increased from 5/40 to 32/40, speech input buffer increased from 1/24 to 6/24, speech input lexicon increased from 3/24 to 12/24, auditory word-picture matching increased from 13/40 to 32/40, and auditory semantic knowledge increased from 0/36 to 20/36. Environmental sound recognition remained at ceiling (23/24 at baseline vs. 24/24 after treatment). In the repetition domain, word repetition improved from 0/30 to 10/30, and word repetition with oral-cue prompting from 6/30 to 15/30. In the writing domain, dictation improved from 0/40 to 17/40. The remaining subtests were at or near ceiling at baseline and remained stable after treatment, including reading, naming, oral reading, picture-based writing, and direct copying.

## Discussion

To our knowledge, this is the first case report to evaluate a combined protocol of high-frequency rTMS over the bilateral STG combined with auditory-speech training in a patient with PWD with bilateral temporal ischemic strokes. After a 10-day intervention, the patient’s WAB Aphasia Quotient increased from 40.8 to 65.9, and her Cortical Quotient rose from 56.47 to 75.73. On PACA, improvements were observed in phoneme discrimination, minimal difference word judgment, speech input buffer, speech input lexicon, auditory word-picture matching, auditory semantic knowledge, word repetition, word repetition with oral-cue prompting, and dictation, while environmental sound recognition, reading, naming, oral reading, picture-based writing, and direct copying remained at ceiling. The patient’s abbreviated Token Test score increased from 3.5/36 to 16/36, reflecting a shift from a profound to a severe level of auditory comprehension impairment.

The use of high-frequency rTMS over the bilateral STG is based on the pathophysiology of PWD, which is a bilateral auditory-phonological disconnection syndrome rather than a central language deficit. According to the dual-stream model, speech perception relies on bilateral STG networks. The left STG supports phonemic decoding and the right STG processes talker-specific phonetic variability (Hickok and Poeppel, 2007; Luthra, 2021). Bilateral STG lesions isolate Wernicke’s area from phonetic input while sparing its internal language operations, thus preserving fluent spontaneous speech (Geschwind, 1965; Poeppel, 2001; Stefanatos et al., 2005). This contrasts with Wernicke’s aphasia, where left posterior STG damage disrupts the phonological-semantic interface (Miceli and Caccia, 2022; Hickok and Poeppel, 2007). In our patient, preserved environmental sound perception indicated that Heschl’s gyrus was relatively spared, localising the critical deficit to phonetic analysis within the STG rather than to early acoustic registration. The goal of rTMS in this setting is thought to be the modulation of excitability of residual neural populations within the bilateral STG, specifically at the level of phonetic and phonological encoding, that is, the spectrotemporal to phonetic transformation stage. We do not interpret the observed gains as reflecting isolated STG specific modulation, because tasks such as auditory word-picture matching, semantic knowledge, repetition, dictation, and the Token Test likely engage broader auditory-language networks including the superior temporal sulcus, the middle temporal gyrus, and their connections. Despite bilateral STG lesions, perilesional and contralateral homologous areas may retain some capacity for processing spectrotemporal features of speech. High-frequency rTMS may lower the activation threshold of these residual networks, potentially making them more responsive to concurrent behavioural training through rTMS induced metaplasticity. We therefore applied bilateral stimulation to maximise engagement of viable ventral-stream networks in both hemispheres. This approach was consistent with evidence that bilateral protocols may yield superior language outcomes when the left hemisphere is severely compromised, even though most previous studies have targeted frontal language areas (Khedr et al., 2014).

The pattern of improvement on the PACA was consistent with the hypothesis that the intervention was associated with enhanced prelexical auditory processing. However, we acknowledge that the observed gains in auditory semantic knowledge, repetition, dictation, and Token Test performance may also reflect changes in auditory working memory, lexical access, attention, task familiarity, or compensatory strategy use. These improvements likely engage a broader auditory-language network beyond the STG, including perilesional cortex, contralateral homologous regions, and lexical-semantic pathways, rather than reflecting isolated STG-specific modulation. At baseline, the patient scored near zero on tasks requiring phonetic analysis, lexical access from auditory input, and auditory-verbal short-term memory (phoneme discrimination, speech input buffer, speech input lexicon, auditory word-picture matching, auditory semantic knowledge, and word repetition), yet performed at ceiling on tasks that rely on visual or written input (visual word-picture matching, oral picture naming, word reading aloud). This dissociative pattern is characteristic of a prelexical auditory processing disruption—that is, a deficit in mapping acoustic-phonetic information onto stored phonological word forms rather than a generalized semantic or lexical access impairment (Poeppel, 2001; Stefanatos et al., 2005). After treatment, significant gains were observed precisely in these early auditory processing modules, whereas visually mediated language functions remained stable. Although the precise mechanisms by which rTMS facilitated these improvements cannot be determined from a single case, high-frequency rTMS is known to induce long-term potentiation-like plasticity and may enhance local cortical excitability and functional connectivity within stimulated networks (Lefaucheur et al., 2014). In a bilateral STG lesion model, 10 Hz rTMS might temporarily increase the responsiveness of residual auditory cortex to speech signals. When combined with structured auditory-speech training, this heightened excitability may strengthen coupling between residual phonetic regions and the preserved lexical-semantic system, thereby facilitating behavioral gains. In the absence of neurophysiological or functional neuroimaging data, these proposed mechanisms remain speculative and should be interpreted as hypotheses.

As auditory-phonetic analysis improved, the patient may have regained the ability to monitor her own speech output and to benefit from auditory feedback during naming attempts, which is consistent with the interactive activation model of lexical access (Dell et al., 1997). Her abbreviated Token Test score increased from 3.5/36 to 16/36, moving from a profound to a severe level of auditory comprehension impairment (De Renzi and Faglioni, 1978). This change, while still reflecting considerable residual deficit, provides ecologically valid evidence that the gains observed on structured PACA tasks translated into improved comprehension of spoken commands in a more naturalistic context. The Token Test imposes heavy demands on auditory-verbal working memory and syntactic processing, functions that are critically dependent on the integrity of the auditory input pathway. The observed improvement suggested that the combined intervention was associated with gains in not only low-level phonetic discrimination but also higher-level processes involved in retaining and manipulating auditory-verbal information.

Only one prior case report has described rTMS for PWD. Luo and Xu (Luo and Xu, 2021) applied 4 Hz rTMS over the left temporal lobe in a patient with unilateral left temporal infarction and reported improvements in auditory comprehension and repetition after 14 sessions, but without concurrent auditory-speech training. Our group previously reported a patient with auditory agnosia following bilateral temporal lobe infarction (Wang et al., 2010). After intervention of conventional speech-language therapy alone, minimal improvement was observed in that case. Compared with Luo and Xu (Luo and Xu, 2021), we employed a higher stimulation frequency (10 Hz vs. 4 Hz) and a bilateral rather than unilateral protocol in this case report, and we combined rTMS with auditory-speech training administered between the two stimulation sessions, thereby pairing neuromodulation with behaviourally relevant auditory input. Compared with our own previous report (Wang et al., 2010), we have replaced conventional speech-language therapy alone with a combined protocol of bilateral rTMS and concurrent auditory-speech training. This combined approach, already advocated in the post-stroke aphasia literature as a means of synergistically enhancing neuroplasticity (Crosson et al., 2019), was therefore implemented. The contrast between our positive outcomes and the limited improvement with conventional training alone in our previous auditory agnosia case (Wang et al., 2010) further suggests the potential added value of rTMS in this challenging population, although this interpretation remains speculative.

Several limitations should be acknowledged. First, as a single case report, the findings are not generalizable. Second, we cannot exclude the possibility that the improvements partly reflect a delayed response to auditory-speech training, given that the patient had received no speech-language therapy for over 3 years. Third, the absence of a sham control or comparative condition precludes conclusions about the relative contribution of bilateral versus unilateral stimulation. Fourth, no follow-up data were available for this case, as the patient was referred from another institution and returned to her local provider after the intervention. Therefore, the durability of the observed gains remains unknown. Fifth, because the intervention combined rTMS with auditory-speech training, we cannot determine the relative contribution of each component. Practice effects also cannot be completely ruled out, as no alternate forms were used for the PACA or Token Test. However, the dissociation between improved auditory tasks and stable visual tasks argues against a generalized practice effect, and training stimuli were drawn from separate lists distinct from assessment materials. Sixth, the stimulation sites were localized using the 10–20 EEG system rather than MRI-guided neuronavigation. As noted by Herwig et al. (2003), the C3/T3 midpoint may not precisely correspond to Brodmann area 22, and the actual stimulated regions may have differed from the intended targets given anatomical variability and the patient’s bilateral lesions. This limits attribution of improvements specifically to STG modulation.

This case provided preliminary evidence that combined high-frequency rTMS over the bilateral STG and auditory-speech training may offer a means to modulate residual auditory networks, leading to improved auditory comprehension and repetition in PWD with bilateral temporal lobe lesions. The pattern of PACA improvements was consistent with the hypothesis that the intervention enhanced the efficacy of residual prelexical processing pathways. Recent evidence have supported the role of rTMS and theta-burst stimulation in improving auditory comprehension after stroke (Jing et al., 2025; Xie et al., 2025). Controlled studies are warranted to confirm these findings and to optimize stimulation parameters for this rare and disabling condition.

## Data Availability

The original contributions presented in the study are included in the article/Supplementary material, further inquiries can be directed to the corresponding authors.
