# Supplementary material for: rTMS over bilateral superior temporal gyri combined with auditory-speech training for post-stroke pure word deafness: a case report
Source: Front Psychol. 2026 Aug 10;17:1911332. doi: 10.3389/fpsyg.2026.1911332 (PMC13530972; doi:10.3389/fpsyg.2026.1911332)
Supplement: Supplementary file 1 [file Table_1.DOCX]

**Supplementary Table S1. Representative examples of each PACA domain assessed in this study.**

| **Domain** | **Subtest** | **Example Stimuli** | **Patient Response** |
| --- | --- | --- | --- |
| **Auditory Comprehension** | Phoneme Discrimination | Pairs of syllables differing in initial consonant (b vs p), final (e vs e), or lexical tone (b1 vs b2); patient judges same/different. | Verbal or button press |
|  | Minimal Difference Word Judgment | Minimal pairs of real words differing in tone (ban1 vs ban4; ma1 vs ma3); patient judges same/different. | Verbal or button press |
|  | Speech Input Buffer | Pairs of digit sequences of increasing length (2 to 7 digits) are presented auditorily (e.g., 24 vs 24 for same, 58 vs 48 for different); patient judges whether the two sequences are identical. | Yes/No button press |
|  | Speech Input Lexicon | Spoken words and nonwords varying in frequency and imageability (e.g., real word “refrigerator,” nonword “box-nail”); patient judges whether each is a real Chinese word. | Yes/No button press |
|  | Environmental Sound Recognition | Common non-speech sounds (e.g., car horn, dog barking, telephone ringing); patient points to corresponding picture. | Point to corresponding picture |
|  | Auditory Word–Picture Matching | Spoken words from semantic categories (objects, plants, animals, body parts; high and low frequency); patient selects matching picture from an array. | Point to corresponding picture |
|  | Auditory Semantic Knowledge | Pictures are presented; for each, yes/no questions are asked auditorily (e.g., show a frog, ask "Can it swim?"); patient answers yes/no. | Yes/No response |
| **Reading** | Visual Word–Picture Matching | Written words from the same semantic categories as the auditory version; patient selects matching picture. | Point to corresponding picture |
|  | Visual Semantic Knowledge | Same semantic questions as the auditory version, presented visually as written sentences; patient answers yes/no. | Yes/No response |
|  | Word Categorization | A list of written words is presented; patient sorts them into semantic categories (e.g., animals, furniture, food). | Verbal or pointing |
| **Naming** | Picture Categorization | A set of pictures is presented; patient sorts them into semantic categories. | Verbal or pointing |
|  | Oral Picture Naming | Pictures of objects, plants, animals, and body parts (high and low frequency) are presented one at a time; patient names each aloud. | Verbal naming |
| **Repetition** | Word Repetition | Monosyllabic, disyllabic, and trisyllabic words are presented auditorily (e.g., “umbrella,” “cotton-padded clothes,” “tractor”); patient repeats each aloud. | Verbal repetition |
|  | Word Repetition with Oral‑Cue Prompting | Same words as above; examiner provides mouth-shape cues to facilitate repetition. | Verbal repetition |
| **Oral Reading** | Word Reading Aloud | Written words varying in imageability and frequency (high image-high frequency, low image-high frequency, high image-low frequency, low image-low frequency); patient reads each aloud. | Reading aloud |
| **Writing** | Dictation | Words from semantic categories (objects, plants, animals, body parts; high and low frequency) are presented auditorily; patient writes each down. | Writing |
|  | Picture‑Based Writing | Pictures from the same semantic categories are presented; patient writes the corresponding word for each. | Writing |
|  | Direct Copying | Written words (high and low frequency) are presented; patient copies each exactly. | Copying |

Abbreviations:PACA, Psycholinguistic Assessment in Chinese Aphasia. Tone numbers: 1 = high level, 2 = rising, 3 = falling-rising, 4 = falling. Examples are romanized or translated for illustrative purposes; original testing was conducted in Chinese using spoken syllables and written characters.
