# Supplementary material for: rTMS over bilateral superior temporal gyri combined with auditory-speech training for post-stroke pure word deafness: a case report
Source: Front Psychol. 2026 Aug 10;17:1911332. doi: 10.3389/fpsyg.2026.1911332 (PMC13530972; doi:10.3389/fpsyg.2026.1911332)
Supplement: Supplementary file 2 [file Table_2.DOCX]

**Supplementary Table S2. Representative examples of tasks used at each hierarchical level of auditory-speech training.**

| **Hierarchical Level** | **Task Type** | **Example Stimuli** | **Task Description** | **Patient Response** | **Difficulty Adjustment** |
| --- | --- | --- | --- | --- | --- |
| **Auditory Detection** | Sound awareness | Pure tones (250Hz, 500Hz, 1000Hz), environmental sounds (doorbell, rain, footsteps), animal sounds (cat meowing, rooster crowing), speech sounds (/a/, /i/, /u/) | Stimuli presented at gradually decreasing intensities (100% → 20% volume); patient responds when sound is heard | Button press or verbal "yes" | Intensity reduces after 3 consecutive correct; increases after 2 errors |
|  | Sound localization | Sounds presented from left, right, or center speaker | Patient identifies direction of sound source | Point to left/right/center | Add background noise (white noise, 0–50% volume) after 5 consecutive correct |
| **Auditory Discrimination** | Same/different judgment | Pairs of vowels (/a/ vs /o/, /i/ vs /u/), consonants (/s/ vs /z/, /t/ vs /d/), tonal contrasts using nonwords | Patient judges whether two auditorily presented sounds are the same or different | Verbal "same" or "different"; or button press | Increase inter-stimulus interval (500ms → 2000ms) after 5 consecutive correct; decrease if 2 errors |
|  | Loudness discrimination | Pairs of tones differing in intensity (50dB vs 60dB, 60dB vs 70dB) | Patient identifies which sound is louder | Verbal "first" or "second"; or button press | Reduce intensity difference (10dB → 5dB → 3dB) after 3 consecutive correct |
|  | Duration discrimination | Pairs of tones with varying duration (500ms vs 1000ms, 1000ms vs 1500ms) | Patient identifies which sound is longer | Verbal "first" or "second"; or button press | Reduce duration difference after 3 consecutive correct |
| **Auditory Identification** | Word–picture matching | Words from daily living categories: tableware: plate, chopsticks; furniture: table, wardrobe; vehicles: car, train; food: noodles, dumplings | Patient hears a spoken word and selects the corresponding picture from an array of 4 | Point to corresponding picture | Increase array size (4→6→8) after 5 consecutive correct; reduce if 2 errors |
|  | Environmental sound identification | Sounds: vacuum cleaner, chainsaw, typewriter, buzzing bee, frying oil | Patient hears a sound and selects corresponding picture | Point to corresponding picture | Increase number of options (4→6) after 5 consecutive correct |
|  | Syllable identification | Spoken syllables (e.g., /da/, /ga/, /ka/, /ta/) with accompanying pictures of objects with similar names | Patient identifies which of 4 syllables was heard by selecting the corresponding picture | Point to corresponding picture | Add foils differing only in one phonetic feature after 5 consecutive correct |
| **Auditory Comprehension** | Yes/No questions | Pictures presented with questions, e.g., show picture of "cat" → ask "Is this a bird?"; show picture of "apple" → ask "Is this a fruit?" | Patient answers yes/no questions based on pictures | Yes/No verbal response or button press | Increase question complexity (simple → compound, e.g., "Is this a fruit and can you eat it?") |
|  | Multi-step commands | Instructions: "Point to the picture of the bird first, then the flower"; "Show me the spoon and then the knife" | Patient follows multi-step auditory commands | Pointing or verbal response | Increase number of steps (1→2→3→4) after consecutive correct responses |
|  | Semantic verification | Auditory sentences: "Cats are birds" (false); "Apples are fruits" (true); "Dogs can fly" (false) | Patient judges whether the sentence is true or false | Yes/No verbal response or button press | Use more abstract statements (e.g., "Honesty is a valuable trait") after 5 consecutive correct |
|  | Story comprehension | Short 2-3 sentence stories presented auditorily, followed by comprehension questions (e.g., "Who helped the boy?", "What color was the bird?") | Patient answers questions about the story | Verbal response or picture selection | Increase story length (2 sentences → 4 sentences) after consecutive correct; repeat questions if errors |
